# Loss of NFE2L3 protects against inflammation-induced colorectal cancer through modulation of the tumor microenvironment

**DOI:** 10.1038/s41388-022-02192-2

**Published:** 2022-01-28

**Authors:** James Saliba, Baptiste Coutaud, Kiran Makhani, Noam Epstein Roth, Jennie Jackson, Joo Yeoun Park, Natascha Gagnon, Paolo Costa, Thiviya Jeyakumar, Marina Bury, Nicole Beauchemin, Koren K. Mann, Volker Blank

**Affiliations:** 1 Lady Davis Institute for Medical Research, Montreal, Canada; 2 Department of Medicine, McGill University, Montreal, Quebec Canada; 3 Goodman Cancer Institute and Departments of Oncology, Biochemistry and Medicine, McGill University, Montreal, Quebec Canada; 4 Department of Pharmacology and Therapeutics, McGill University, Montreal, Quebec Canada; 5 Department of Physiology, McGill University, Montreal, Quebec Canada; 6 Present Address: Life Sciences Institute and Department of Microbiology and Immunology, University of British Columbia, Vancouver, British Columbia Canada; 7 Present Address: De Duve Institute, UCLouvain, Brussels, Belgium

**Keywords:** Colorectal cancer, Prognostic markers, Inflammation, Cytokines, Sequencing

## Abstract

We investigated the role of the NFE2L3 transcription factor in inflammation-induced colorectal cancer. Our studies revealed that *Nfe2l3*^−/−^ mice exhibit significantly less inflammation in the colon, reduced tumor size and numbers, and skewed localization of tumors with a more pronounced decrease of tumors in the distal colon. CIBERSORT analysis of RNA-seq data from normal and tumor tissue predicted a reduction in mast cells in *Nfe2l3*^−/−^ animals, which was confirmed by toluidine blue staining. Concomitantly, the transcript levels of *Il33* and *Rab27a*, both important regulators of mast cells, were reduced and increased, respectively, in the colorectal tumors of *Nfe2l3*^−/−^ mice. Furthermore, we validated NFE2L3 binding to the regulatory sequences of the *IL33* and *RAB27A* loci in human colorectal carcinoma cells. Using digital spatial profiling, we found that *Nfe2l3*^−/−^ mice presented elevated FOXP3 and immune checkpoint markers CTLA4, TIM3, and LAG3, suggesting an increase in Treg counts. Staining for CD3 and FOXP3 confirmed a significant increase in immunosuppressive Tregs in the colon of *Nfe2l3*^−/−^ animals. Also, Human Microbiome Project (HMP2) data showed that NFE2L3 transcript levels are higher in the rectum of ulcerative colitis patients. The observed changes in the tumor microenvironment provide new insights into the molecular differences regarding colon cancer sidedness. This may be exploited for the treatment of early-onset colorectal cancer as this emerging subtype primarily displays distal/left-sided tumors.

## Introduction

Despite clinical advances in cancer diagnosis and treatment, colorectal cancer (CRC), the third most diagnosed malignancy, is still the fourth leading cause of cancer mortality worldwide [1]. While CRC incidence is declining among subjects aged 50 years and above, an opposite trend is observed among young adults presenting with early onset CRC [2]. Early onset CRC is characterized by more aggressive tumors predominant in the left/distal colon [3, 4]. There are many risk factors that affect colorectal cancer, including diet, smoking, microbiome composition, and inflammation [5]. Patients with inflammatory bowel diseases (IBDs), such as ulcerative colitis and Crohn’s disease, are at a higher risk of developing CRC and 10–15% of those patients will die from IBD-associated colorectal cancer [6]. Hence, a better understanding of the molecular mechanisms involved in colitis-induced colon tumorigenesis is needed for the development of better diagnostic and therapeutic strategies.

The AOM/DSS model is a murine model commonly used for studying colitis-associated colorectal carcinoma. Azoxymethane (AOM) is a colon carcinogen that induces genetic mutations and treatment with dextran sodium sulfate (DSS) leads to intestinal inflammation and colitis. Combined, both agents mimic IBD-induced CRC [7]. Of interest, when this model was first described, elevated numbers of mast cell counts were observed in the proximity as well as within carcinoma tissue warranting further studies on mast cell involvement in AOM/DSS-induced CRC [8].

Mast cells are activated during inflammatory and allergic reactions leading to their degranulation and the release of a plethora of immune- and tumorigenesis-modulating factors. These factors include histamine and proteases, such as chymase and tryptase able to degrade ECM components and promote tumor cell implantation, as well as angiogenic and growth/differentiation factors, such as VEGF and IL8, respectively [9]. Mast cell activation is accompanied by the recruitment of additional mast cell progenitors to the site of inflammation leading to an increase in mast cell numbers [10]. Multiple proteins control mast cell activation or have been linked to the function of this cell type, including the RAS oncogene family member RAB27A and interleukin 33 (IL33). IL33 is an alarmin cytokine that is released in response to cell injury, infection, or mechanical damage, to induce inflammation [11] through the promotion of mast cells granulation and recruitment to the site of injury [12]. RAB27A inhibits mast cell degranulation and activation [13]. In mice, the role of mast cells in CRC has been controversial as they can either be linked to anti- or pro-tumor properties depending on the colitis stage. During early inflammation stages, mast cells localize in areas promoting mucosal healing and reduction of inflammation. However, in later stages when adjacent to transformed cells, they exert pro-tumor activity since tumors with a high number of mast cells are less differentiated and more aggressive [14]. Thus, a better knowledge of mast cell recruitment is key to understand how colitis induces CRC.

NFE2L3 has been linked to several types of cancers in humans, including colon, breast, thyroid, pancreas, and kidney [15–20]. *NFE2L3* was also identified as one of the most significantly mutated genes in tumors across 12 cancer types [15, 21]. Recently, it was shown that knockdown of *NFE2L3* reduced human colon cancer cell proliferation through an increase in the levels of DUX4, a protein that functions as a CDK1 inhibitor [22]. It was also found that the β-catenin/TCF4 complex of the Wnt signaling pathway activates the expression of NFE2L3 transcripts in colon as well as other cancer cells [23]. In the mouse, the *Nfe2l3* gene has been shown to be significantly upregulated in the colonic mucosa of mice following AOM/DSS treatment [24], yet its function in inflammation-induced colon cancer has not been investigated.

Here, we analyzed the role of NFE2L3 in tumor development in vivo using a mouse model of colitis-associated CRC, based on the treatment with the carcinogen azoxymethane and the inflammation-inducing agent dextran sodium sulfate [8, 25]. We found that *Nfe2l3*^−/−^ animals are protected against CRC, developing significantly less tumors than *Nfe2l3* heterozygote and wild type mice. We showed that loss of NFE2L3 leads to less inflammation and a change in the tumor microenvironment, with a reduced number of mast cells and an increase in regulatory T cells, mediated through signaling via the IL33 and RAB pathways.

## Results

### NFE2L3 promotes inflammation and colitis-associated tumorigenesis

**Fig. 1:**
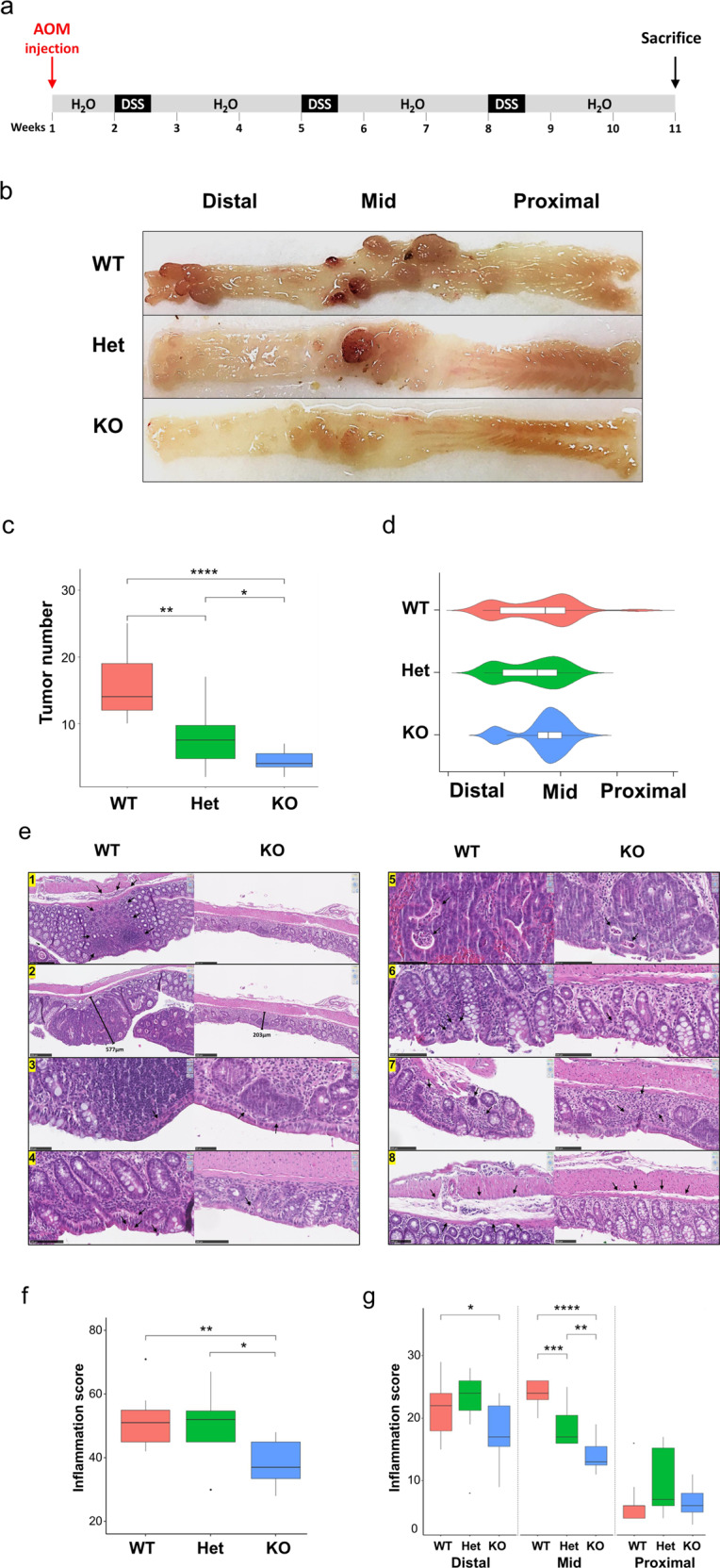
Loss of NFE2L3 leads to a decrease in tumor burden and inflammation. **a** AOM was injected at day 1 at 7 mg/kg. Starting at day 8, three repeated treatments of 2.5% DSS were given with 4 days of DSS administration followed by 17 days of water. Mice were sacrificed at day 70 and 71. **b** Schematic depicting the digestive track and the different segments of the colon along with representative pictures of AOM/DSS mouse colons opened longitudinally from littermates with the genotypes *Nfe2l3*^+/+^ (WT), *Nfe2l3*^*+/−*^ (Het) and *Nfe2l3*^*−/−*^ (KO). **c** Boxplot representation of tumor number distribution per genotype. Tumors were counted from wild type, heterozygous and knockout mice (*n* = 9–12). **d** Violin plot with imbedded boxplot representation of tumor distribution pattern. Colon length was normalized from 0 to 100 and sections were split into three equal parts distal (0–33.3), mid (33.4–66.6) and proximal (66.7–100) colon. **e** Hematoxylin/Eosin stained sagittal sections of wild type and knockout colons. Criteria observed are inflammatory cell infiltration and cell depth (1), mucosal thickening (2), surface epithelial degeneration (3), gland epithelial apoptosis (4), gland degeneration/abscesses (5), goblet/enterocyte ratio decrease (6), gland loss (7), and submucosal edema (8). Boxplot representation of total colon inflammation per genotype in full length (**f**), or distal, mid, and proximal sections (**g**), scored from 0 to 4 using the above criteria. Data represent unpaired *t-*test **c–g**; unpaired *t-*test; *p ≤ 0.05; **p ≤ 0.01; ***p ≤ 0.001 and ****p ≤ 0.0001. Black dots represent outliers.

To determine NFE2L3 function during colitis-associated carcinogenesis, we subjected adult, 8–14-week-old wild type, *Nfe2l3*^+/–^ or *Nfe2l3*^−/−^ mice to the well-established AOM/DSS model (Fig. 1a, b) [26]. The number, as well as the size of colon tumors, was significantly lower in *Nfe2l3*^−/−^ mice than in wild type animals while *Nfe2l3* heterozygote mice showed an intermediate phenotype, hinting at a dose effect of the transcription factor (Fig. 1c, Supp. Fig. 1a). Colons from all three genotypes displayed frequent tumors in their distal and mid sections but few to no tumors in their proximal sections (Fig. 1b, d). *Nfe2l3*^−/−^ tumors were significantly enriched in the mid section of the colon (87%) compared to the other genotypes where tumors were more evenly distributed between the distal (45%) and mid sections (55%), suggesting location-based differences in tumor signaling. Only wild type mice displayed a small number (3.5%) of tumors in the proximal part of the colon (Fig. 1d, Supp. Fig. 1b). To assess the inflammatory response, we used nine histopathological criteria and scored inflammation and tissue injury (Fig. 1e), as described previously [27]. A board-certified pathologist blindly scored hematoxylin/eosin-stained sections from wild type and knockout colons for each criterion from 0 to 4 (0-absence; 1-very mild/debatable; 2-mild; 3-moderate; 4-severe). Compared to wild type tumors, inflammation was significantly lower in knockout tumors in both the mid and distal sections of the colon with a much more prominent decrease in the mid section. Inflammation in *Nfe2l3*^+/–^ mice however was only significantly lower in the mid section. Little inflammation was observed in the proximal colon (Fig. 1f, g). With respect to tissue injury, *Nfe2l3*^−/−^ mice scored substantially lower than wild type animals, with the observed effect being only significant in the mid section of the colon (Supp. Fig. 1c, d). To validate our findings in humans, we analyzed RNA-seq data from the Human Microbiome Project (HMP2), revealing that NFE2L3 transcript levels are significantly elevated in the rectum of patients with ulcerative colitis, with a trend increase also observed in the colon, but no change was found in the lower intestine (Supp. Fig. 2) [28]. Together, these data strongly suggest a location- and genotype-dependent role of NFE2L3 in the modulation of inflammation and tumor development in our CRC model.

### NFE2L3 deficiency disrupts mast cell homeostasis in colonic tissue

We found that colonic inflammation was less severe in *Nfe2l3* knockout mice. To identify the immune infiltrating cells conferring this phenotype, we collected RNA from both normal and tumor tissue samples of all genotypes and performed RNA-seq analyses. We interrogated these data using CIBERSORT, a tool that deconvolutes RNA-seq results using a signature matrix to profile the distribution of immune cell types in mixed cell populations [29]. Among the immune cells profiled, only significant changes between genotypes and/or tissue states (tumor vs normal) were considered.

**Fig. 2:**
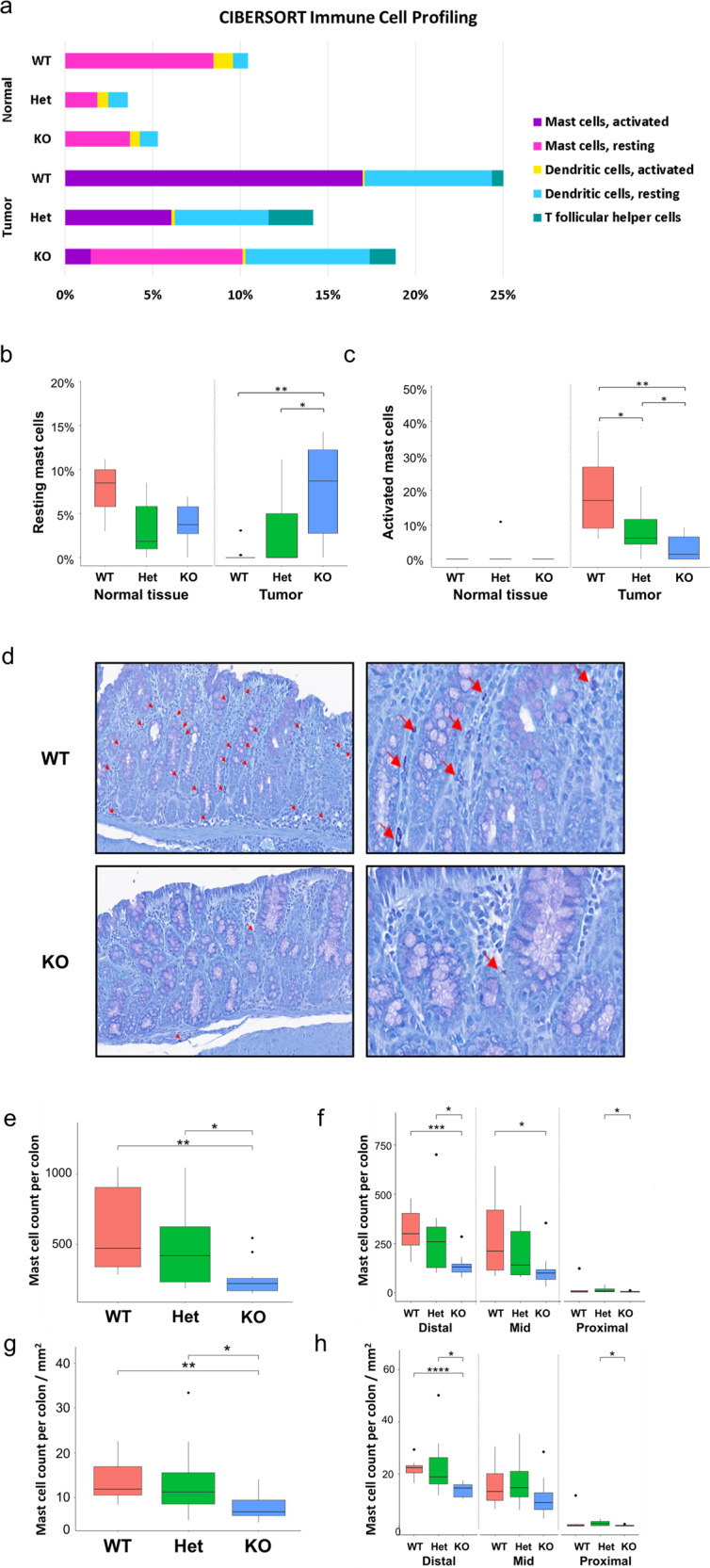
NFE2L3 deficiency results in a decrease in mast cell numbers and activation within tumors. **a** Percent stacked bar plot representation of estimated abundance of the significantly altered immune cell types computed using CIBERSORT. **b**, **c** Relative abundance of resting and activated mast cells from normal tissues and tumors. *t-*test was performed for statistical analysis. **d** Toluidine blue-stained sagittal sections of colons from WT and KO mice, mast cells are indicated with red arrows. **e**, **f** Average mast cell number per genotype or **g**, **h** mast cell number per mm^2^ for the full-length colon, as well as the distal, mid and proximal colon sections. Data represent unpaired t-test; *p ≤ 0.05; **p ≤ 0.01; ***p ≤ 0.001 and ****p ≤ 0.0001. Black dots represent outliers.

Our analysis showed that all tumor samples were enriched with resting dendritic cells compared to normal specimens, which had significantly more activated dendritic cells but fewer dendritic cells in total. Furthermore, we found that T follicular helper (Tfh) cells infiltrated tumor samples, whereas no Tfh cell signal was detected within the normal tissue. Finally, tumor samples exhibited significantly more mast cell infiltrates that also comprised more activated mast cells when compared to normal tissue (Fig. 2a).

With respect to differences between genotypes, only changes in mast cells were significant among the assessed immune cell types. Within the normal tissue, wild type samples tended to be more infiltrated with resting mast cells compared to knockout specimens (Fig. 2b). In tumor samples, *Nfe2l3* knockout mice displayed significantly more resting mast cells in comparison to wild type animals that in turn exhibited significantly more activated mast cells (Fig. 2b, c). To validate our findings, we stained the colon tissues with toluidine blue dye to examine mast cell infiltration (Fig. 2d) and indeed detected a lower number of mast cells in the colon tissue of *Nfe2l3*^−/−^ mice, with the distal colon being the most significantly affected section (Fig. 2e, f). To account for potential tissue size differences, we normalized mast cell counts by colon tissue size and confirmed that the mast cell count was significantly lower in knockout tissue, most particularly in the distal section of the colon. *Nfe2l3*^+/−^ mice presented an intermediate phenotype in both cases (Fig. 2g, h). Our results suggest that the presence of NFE2L3 impacts inflammation and tumorigenesis by increasing the number of activating mast cells during chronic inflammation in a spatial- and genotype-dependent manner.

### NFE2L3 modulates *IL33* and *RAB* and RAB effector gene expression

**Fig. 3:**
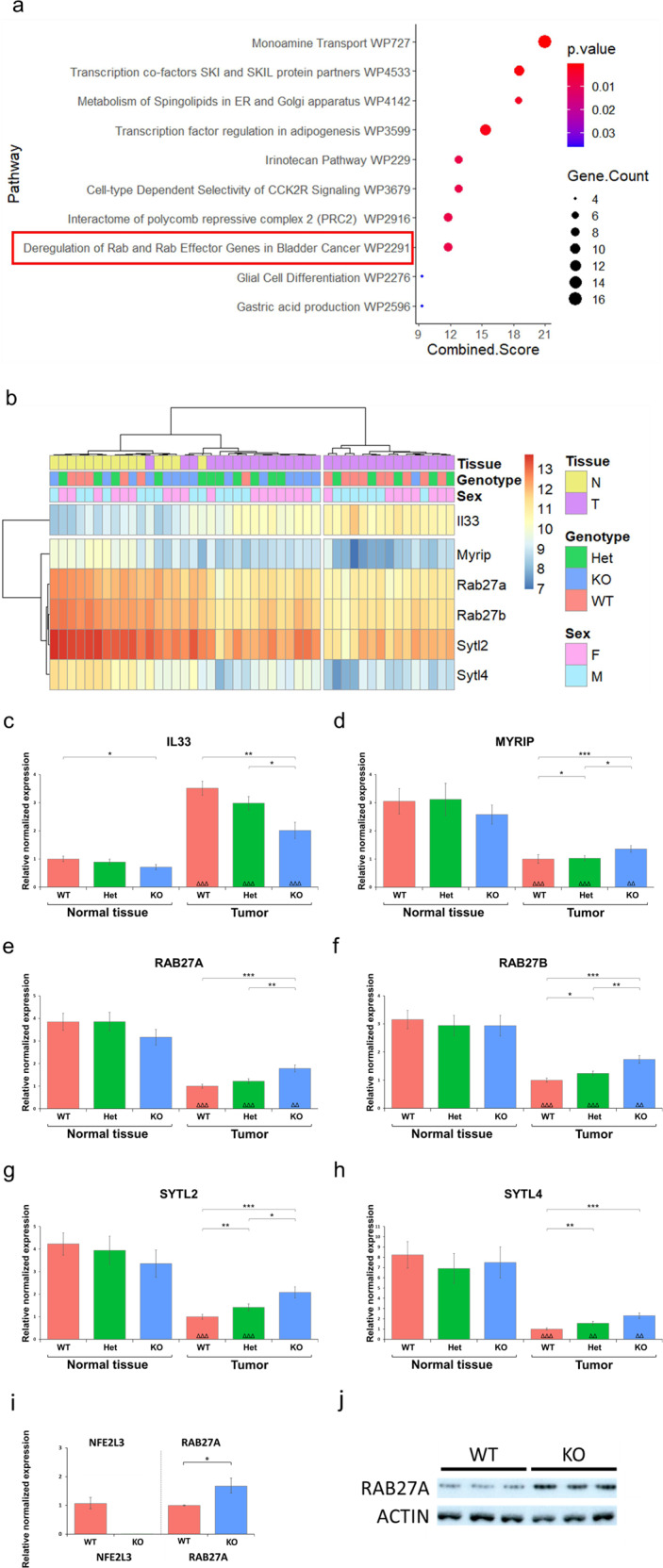
Loss of NFE2L3 leads to the downregulation of *Il33* mRNA and upregulation of transcripts linked to the RAB pathway. **a** Top 10 pathways resulting from Enrichr [79] enrichment analysis. **b** Heatmap of differentially expressed genes of the wikipathway “Deregulation of RAB and RAB Effector Genes in Bladder Cancer_Homo sapiens_WP2291” comprising the *Sytl2*, *Rab27a*, *Rab27b*, *Sytl4,* and *Myrip* genes [76] as well as *Il33*. **c–h** RT-qPCR expression analysis of genes shown in the heatmap (**b**). **i** RT-qPCR expression analysis of *Nfe2l3* and *Rab27a* in NFE2L3 wild type and knockout BMMCs (*n* = 3 in triplicates). *Eef2*, *Rplp0*, *Nono*, and *Tbp* were used as reference genes. **j** Immunoblot analysis of NFE2L3 wild type and knockout BMMCs using RAB27A antibody (Abcam, ab55667) (*n* = 3 in triplicates). Actin was used as loading control. Data represents normalized relative expression values ± SD; Unpaired *t-*test between genotypes is represented in *p ≤ 0.05; **p ≤ 0.01; ***p ≤ 0.001 and ****p ≤ 0.0001, and between normal tissue and tumor within the same genotype; ^ΔΔ^*p* ≤ 0.01 and ^ΔΔΔ^*p* ≤ 0.001.

Based on these data, we carried out studies to identify the molecular mechanisms involved in the modulation of inflammation, mast cell recruitment and tumor progression in our AOM/DSS-treated mice. Pathway enrichment analysis of our RNA-seq data of tumor tissue derived from the different genotypes revealed that the deregulation of RAB and RAB effector pathway was one of the top 10 most enriched pathways in knockout tumors when compared to wild type (Fig. 3a, Table S1). This is of interest as members of the RAB pathway differentially control mast cell degranulation [13]. RNA-seq also revealed that *IL33* transcript levels were significantly increased in all tumor samples regardless of the genotype. We further found that tumor samples from *Nfe2l3* knockout animals displayed significantly reduced *IL33* expression when compared to wild type (Fig. 3b). This is relevant, as IL33 has been shown to be a regulator of mast cell activity [30]. Our RT-qPCR analysis of *Il33* and RAB pathway mRNAs confirmed these observations as *Il33* transcripts were significantly increased in all tumors and reduced in knockout samples in both tumor and normal tissues (Fig. 3c). In addition, transcripts of genes belonging to the RAB pathway, including *Rab27a/b*, *Sytl2/4* and *Myrip* were significantly downregulated in all tumors versus normal samples (except for *Sytl2* in *Nfe2l3*^−/−^ samples), as well as elevated in knockout tumors compared to wild type samples (Fig. 3d–h). *Nfe2l3*^+/–^ mice displayed an intermediate phenotype throughout (Fig. 3c–h). Finally, to confirm whether the observed alterations occur within mast cells, we derived bone marrow derived mast cells (BMMCs) from our mice using a previously described protocol [31]. RT-qPCR and immunoblot analysis revealed that BMMCs from *Nfe2l3* knockout mice exhibit increased *Rab27a* transcript as well as RAB27A protein levels, demonstrating that loss of NFE2L3 in mice modulates mast cell activity through RAB27A upregulation (Fig. 3i, j).

### NFE2L3 regulates *IL33* and *RAB27A/B* at the transcriptional level in human HCT116 colorectal cancer cells

**Fig. 4:**
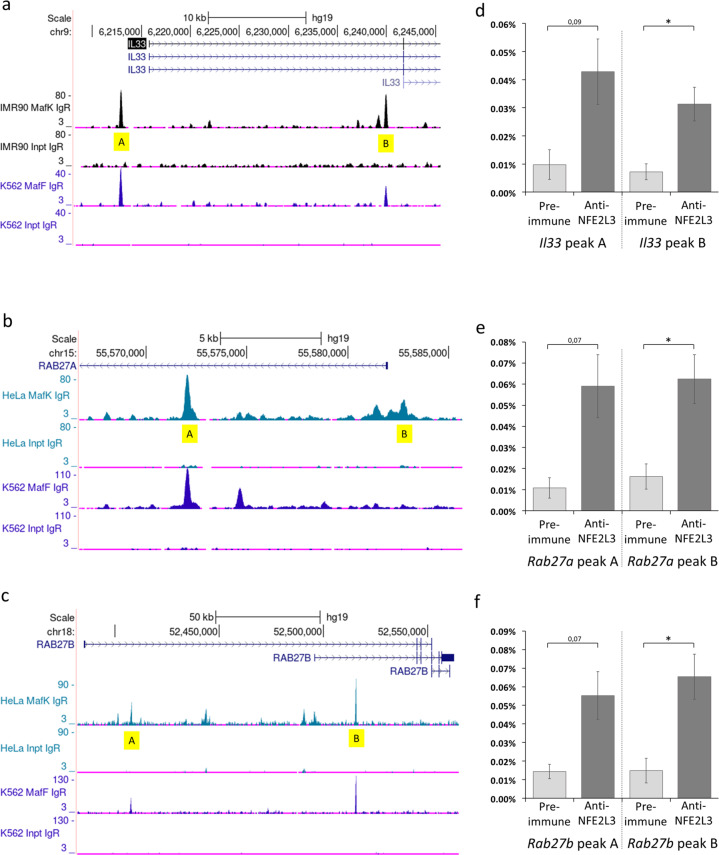
NFE2L3 regulates *Il33* and *Rab27a/b* on a transcriptional level in human CRC HCT116 cell lines. Binding of NFE2L3 transcription factor to IL33 and RAB27A/B loci in HCT116 CRC cells. **a–c** Location of NFE2L3 binding partners, MAFF and MAFK, DNA binding sites at the *IL33* and *RAB27A/B* loci were established using the UCSC genome browser database and data from the ENCODE consortium [32], showing MAFF/K specific binding to *IL33* and *RAB27A/B* loci in HEPG2 cells, respectively. Chosen peaks are marked in yellow. **d–f** Classical ChIP-qPCR analysis of NFE2L3 transcription factor binding to *IL33* and *RAB27A/B* loci. HCT‐116 cells were analyzed by chromatin immunoprecipitation using antibodies against NFE2L3 or Rabbit preimmune serum to control for non‐specific binding. Data represent mean values ± SD; unpaired *t*‐test; *P < 0.05.

To identify whether the regulation of IL33 and RAB27A/B by NFE2L3 occurs at the transcriptional levels and applies to humans, we used public ChIP-seq ENCODE data [32] of NFE2L3 dimerization partners MAFF and MAFK to identify potential NFE2L3 binding sites in the promoter regions of the *Il33*, *Rab27a* and *Rab27b* genes (Fig. 4a–c). As not all MAFF and MAFK binding sites may be bona fide NFE2L3 recognition elements, we analyzed multiple candidate peaks by ChIP-qPCR using a NFE2L3 specific antiserum [22]. We examined human colorectal carcinoma HCT116 cells transfected with an empty construct or a construct expressing the nuclear form of NFE2L3. We found significant binding of NFE2L3 in *Il33*, *Rab27a* and *Rab27b* genes, suggesting similar regulation in human and mouse CRC (Fig. 4d–f).

### NFE2L3 loss alters tumor infiltrating T cell signatures

**Fig. 5:**
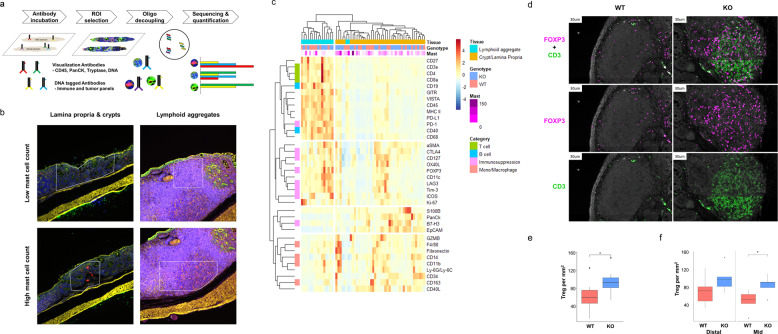
NFE2L3 deficiency promotes an immunosuppressive microenvironment and an increase in Treg count. **a** DSP analysis was performed using tagged antibodies from both the immune and tumor signature panels (Nanostring). Slides were stained with immune-fluorescence visualization antibodies targeting tryptase mast cell marker (Red), CD45 leukocyte marker (Yellow), PanCK cytokeratine marker (Green), and SYTO™ 83 DNA marker (Blue). **b** Highlighted areas represent regions of interest (ROIs) chosen from WT and KO colons to target lamina propria and crypts or lymphoid aggregates with varying numbers of mast cells (red). **c** Heatmap of differentially expressed proteins from WT and KO colons generated using Digital Spatial Profiling technology (Nanostring). Mast cells were counted, and a relative score added to the heatmap in purple to highlight mast cell abundance. **d** Slides were stained with CD3 T-cell marker (green) and FOXP Treg marker (magenta). **e** Relative abundance of Tregs in the full-length colon or **f** distal and mid sections of WT and KO mice (*n* = 5). Data represent unpaired *t-*test; *p ≤ 0.05.

Mast cells modulate the immune response to tissue injury and have been linked to both humoral and cell mediated immunity [9, 33]. To identify changes in the populations of infiltrating immune cells, we employed digital spatial profiling (DSP). DSP recognizes a panel of protein markers from chosen regions of interest (ROIs). Our choice of ROIs consisted of a combination of multiple criteria, including genotype, lymphoid aggregates versus crypts or lamina propria, mast cell counts and tissue state (normal vs tumor) (Fig. 5a, b). Unsupervised clustering analysis of the multitude of ROIs revealed that the two dominant signatures within the tissues resulted from the lymphoid aggregates compared to crypts and lamina propria (Fig. 5c). Clustering revealed that lymphoid aggregates were enriched with B (CD40, CD19) and T (CD3e, CD4, and CD8a) cell specific signatures, while crypts and lamina propria had dendritic, monocyte and macrophage signatures. The signatures of wild type and *Nfe2l3*^−/−^ mice were indistinguishable within the crypt/lamina propria cluster but clustered separately within the lymphoid aggregates. While the lymphoid aggregates of both genotypes exhibited T and B cell infiltration signatures, knockout mouse lymphoid aggregates displayed stronger immune checkpoint and T cell suppression signatures that clustered together and included CTLA4, CD127, FOXP3, LAG3, and TIM3 markers [34, 35]. However, while the PD-1 immune checkpoint signature was enriched within the lymphoid aggregate cluster, this phenotype was not exclusive to knockout animals (Fig. 5c). In summary, our data suggest that the tumor microenvironment in *Nfe2l3*^−/−^ mice is highly immunosuppressive.

We thus investigated whether NFE2L3 deficiency leads to an increase in the immunosuppressive T cell (Treg) count. To assess the levels of Treg infiltration, we stained colon tissues with the T cell marker CD3 and Treg marker FOXP3 by immunofluorescence [36] (Fig. 5d). Quantitative analysis confirmed the presence of a higher number of Tregs in *Nfe2l3*^−/−^ samples when compared to wild type specimens, reaching significance in the colon mid section (Fig. 5e, f). Our results suggest that loss of NFE2L3 leads to an increased presence of immunosuppressive T cells particularly in the mid section of the colon.

## Discussion

The underlying molecular mechanisms of CRC pathology are complex and comprise an array of interactions between various tissues and cell types. Inflammation and modulation of the tumor microenvironment play crucial roles in the progression of colorectal cancer. Thus, a better understanding of how the tumor and its microenvironment are connected is key. Recently, a series of studies linked the NFE2L3 transcription factor to colorectal cancer yet its role in cancer progression remains to be explored [15, 19, 23, 37, 38]. *Nfe2l3* transcripts are elevated in the colonic mucosa in an inflammation-induced cancer model in mice following treatment with AOM/DSS [24]. Furthermore, NFE2L3 levels have been shown to be induced by both TNF and INFG, highlighting its implication in inflammatory processes [39–41]. Therefore, we sought to examine the role of NFE2L3 in inflammation-induced colorectal cancer following treatment with AOM/DSS. Using this model, we discovered that the presence of NFE2L3 promotes colorectal cancer progression through modulation of the tumor microenvironment and enhancing the inflammatory response.

**Fig. 6:**
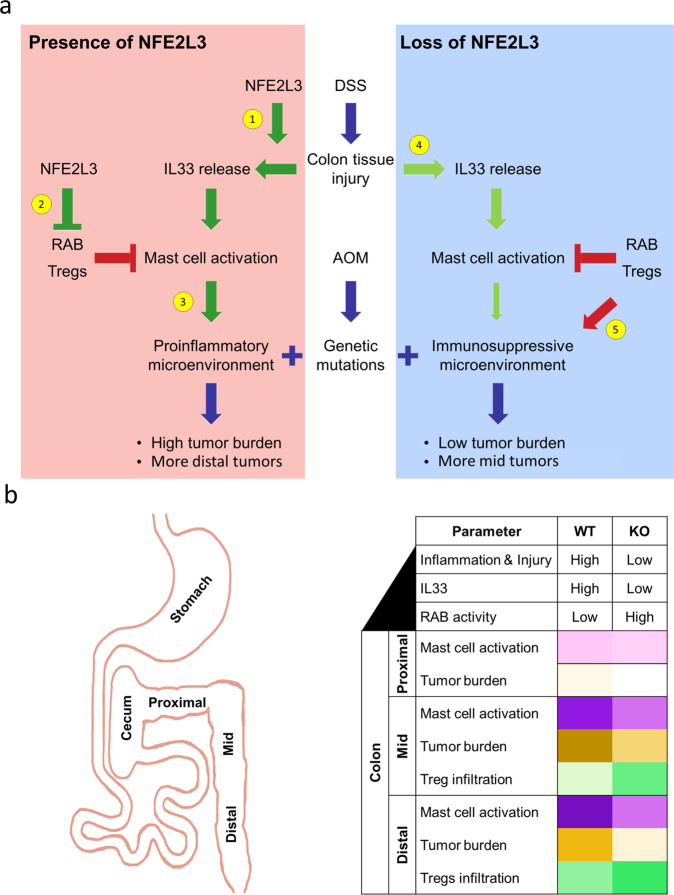
Role of the NFE2L3 transcription factor in colitis-induced CRC. **a** Model of the regulation of CRC development in the presence and absence of NFE2L3. AOM/DSS induces colonic epithelial tissue damage resulting in the release of the alarmin IL33. 1) NFE2L3 promotes *IL33* expression thus leading to mast cell activation; 2) NFE2L3 inhibits Tregs and expression of RAB pathway transcripts hence permitting mast cell activation and degranulation; 3) Elevated mast cell activity led by NFE2L3 expression promotes a proinflammatory microenvironment leading to a higher tumor burden and enrichment of tumors in the distal colon; 4) In absence of NFE2L3, the colonic tissue releases less IL33 in response to injury leading to reduced mast cell recruitment. 5) In the absence of NFE2L3, RAB pathway members and Tregs can inhibit mast cell activity hence promoting an immunosuppressive microenvironment characterized by a lower tumor burden and proximal sided tumors. **b** Table summarizing the differences between wildtype and knockout CRC tissues. The colons of *Nfe2l3* knockout mice display less inflammation and a lower number of tumors when compared to their wild type littermates. Tumors are significantly enriched in the mid versus the distal section of the colon. In addition, *Nfe2l3*^*−/−*^ mice exhibit reduced mast cell infiltration mostly in the distal section of the colon. Finally, knockout mouse colons display significantly higher infiltration by Tregs particularly in the mid section of the colon.

Our CIBERSORT [29] analysis detected an increase in dendritic and mast cells in tumor samples compared to normal. These findings are consistent with previous data showing enrichment of dendritic and mast cells in CRC tumor tissue [14, 42]. With respect to genotype, CIBERSORT uncovered a decrease in activated mast cells in *NFE2L3* knockout mice. We further found that NFE2L3 is required for the induction of *Il33* transcripts and the reduction of mRNA levels of members of the RAB pathway. These results are relevant as the RAB pathway differentially controls mast secretory granules and IL33 activates mast cells by promoting their granulation and recruitment to the site of injury (Fig. 6a) [12, 13].

The role of mast cells during colorectal cancer development has been a matter of debate [43]. However, recent studies have tried to reconcile the different findings in the context of the AOM/DSS model. Mast cells, during initial stages of colitis, play a beneficial role through promotion of tissue repair and subsequent reduction of inflammation. Yet, mast cells adopt a tumor-promoting role when tumors start to develop by enhancing tumor growth and angiogenesis [14, 44–46]. The connection between the alarmin IL33 and mast cells is reciprocal. IL33 is critical to mast cell function as a recent single cell analysis found that IL33 increased both the numbers and magnitude of degranulation in chemokine-producing mast cells [47]. On the other hand, mast cells secrete proteases that cleave IL33, giving rise to a shorter more mature form that is thirty times more active [30]. IL33 is also implicated in CRC tumorigenesis independently of mast cell activity. In vivo, IL33 stimulates human CRC cell proliferation and metastasis through induction of prostaglandin-endoperoxide synthase 2 (PTGS2) and MMP2/9 [48–50]. IL33 is thus a key regulator of inflammation and CRC progression [50].

RAB proteins are RAS-related GTP-binding proteins specialized in membrane trafficking. While RAB27A/B share 70% similarity at the amino acid level, their roles within secretory pathways are distinct. Whereas RAB27A deficiency leads to a hypersecretory mast cell phenotype, RAB27B deficiency impairs mast cell secretion [13]. Knockdown of RAB27A effector *SYTL4* alone was able to mimic loss of RAB27A phenotype [51]. SYTL2 and MYRIP are also associated with RAB27A function by allowing granule docking through their interaction with phosphatidylinositol-4,5-bisphosphate (PIP2), thus linking vesicles to actin filaments respectively [52]. The RAB members are key in the regulation of secretory pathways and CRC. Elevated RAB27A has been identified as a positive prognostic marker of colorectal cancer, whereas a decrease of both RAB27A and RAB27B expression correlates with increased metastasis and poor outcome [53, 54].

In our studies, we showed that in the presence of NFE2L3, the expression of *Rab27a/b* and of *Rab27a* effectors *Sytl2/4* and *Myrib* are all decreased. While *Rab27b* was also elevated, the increase in *Rab27a* effectors implies that the granule secretion balance shifted towards a pro secretion phenotype mediated by RAB27A. This suggests that due to loss of RAB27A activity in the presence of NFE2L3, mast cell degranulation is promoted leading to the potentiation of IL33 activity [23, 30]. Our RT-qPCR analysis revealed that in normal non-tumor tissue, *Il33* transcripts were expressed at significantly lower levels in *Nfe2l3*^−/−^ specimens whereas the RAB pathway genes were unaltered. We thus hypothesize that NFE2L3 increases inflammation by priming the colonic tissue to inflammatory processes through the increase of *IL33* transcripts and subsequent secretion of the cytokine upon injury. Our findings are of relevance for the clinic, as our data show that elevated *Nfe2l3* expression leads to an increase in *Il33* and decrease in *Rab27a* expression, two observations that have been associated with poorer prognosis for CRC in humans [54]. Further experimentation with earlier time points is required to better understand the chronological order of events.

Tregs and mast cells are abundant in CRC and are known to regulate each other. On one hand, mast cells alter Treg function towards a proinflammatory state [33]. On the other hand, Tregs were shown to suppress mast cell degranulation [55].

Our digital spatial profiling analysis revealed a modulation in signaling upon altered NFE2L3 expression. While all lymphocyte aggregates were enriched with B and T cell signatures, *NFE2L3* knockout mice presented elevated FOXP3 and immune checkpoint marker expression as well as elevated Treg counts, all signs of T cell exhaustion [56]. This is of interest as tumors infiltrated with exhausted T cells are considered hot tumors that are responsive to immune checkpoint inhibitor drugs aimed at reactivating the infiltrated T cells [57]. *NFE2L3* levels may thus be a predictor of response to immune checkpoint inhibitor drug therapies targeting immune checkpoints CTLA4, TIM3 and LAG3 [34, 35], with better outcomes potentially coinciding with lower *NFE2L3* expression.

While the frequency of CRC has been on a continuous decline among older individuals, the incidence of CRC among young adults is increasing worldwide, but the leading cause for the latter is unknown [58]. Moreover, tumors among young adults are often detected at a more advanced stage at diagnosis, are less differentiated and are located to the left (or distal) side of the colon [59]. Tumor sidedness, meaning the location of the tumor throughout the colon, is becoming an increasingly important concept [60]. Differences between left and right tumors are well established, with left tumors being less immunogenic compared to right tumors which are highly infiltrated by T cells [61]. In our studies, while loss of NFE2L3 decreased the overall tumor burden, it preferentially did so in the distal section of the colon. NFE2L3 may thus carry out different roles and/or is distinctly regulated depending on its position within the colon. Distally, deficiency of NFE2L3 coincides with a loss of mast cells and a decrease in tumor burden, whereas proximally it promotes Treg infiltration and subsequent reduction of inflammation (Fig. 6b). Given the molecular differences between the proximal and distal parts of the colon and the dual role NFE2L3 plays, it would be of interest to assess whether NFE2L3 can serve as a prognostic factor while taking the position of the tumor into account. This is most relevant with the current emergence of early onset CRC that is mostly characterized by left/distal sided tumors [3].

Altered expression of *NFE2L3* has been linked to cancers of the colon, breast, thyroid, pancreas, kidney, lymphatic system and liver [16–18, 22, 62, 63]. Although elevated *NFE2L3* levels correlated with protective roles during breast cancer and T-cell lymphoma [18, 62], it had the opposite effect in thyroid, pancreas, hepatocellular and colorectal carcinoma [16, 17, 22, 64].

While the mechanism of action was not studied in T-cell lymphoma [62], NFE2L3s protective role in breast cancer was due to its inhibition of cell growth and metastasis by suppression of AKT signaling, MMP expression and epithelial–mesenchymal transition (EMT) [18]. As for its oncogenic role, NFE2L3 has been linked to a multitude of cancer hallmarks, proliferation through modulation of the cell cycle [22], maintaining survival by relieving proteotoxic stress [23], resistance to apoptosis [63] and suppressing migration, invasion, and EMT [64]. Genes with different and opposing roles between tissue types are not uncommon, since every tissue has a distinct gene signature and microenvironment. Even in the same tissue, protein function can be time and context dependant as is the case for IL33, being a tumor suppressor during initial stages of colitis and an oncogene at later stages of the disease [65]. The distinct and sometimes opposing roles of NFE2L3 may possibly be due the formation of complexes with other proteins. NFE2L3 functions as a transcription factor through dimerizing with different members of the small MAF family [41]. Different tissues or tissue compartments may express distinct levels of small MAF proteins, thus possibly altering NFE2L3 targets and function [66]. Another possible mechanism are distinct post-translational modifications in various tissues or at distinct stages of tumor development. In this respect, it has been shown that NFE2L3 is ubiquitinated and targeted for degradation by the tumor suppressor FBXW7 [67] or BTRC [15].

While we established a role for NFE2L3 in the promotion of inflammation-induced colorectal cancer, it is unclear whether the observed phenotype is due to loss of NFE2L3 within the colonic cells and/or the immune cells or both. Our HMP2 findings showed that NFE2L3 transcripts are significantly enriched in the rectum during ulcerative colitis. This suggests that NFE2L3 may be exerting part of its observed effects from within the colonic tissue in the distal section where it is highly expressed while exerting them solely through the surrounding immune cells in the proximal regions. In future studies, to determine whether NFE2L3 exerts its effect though the colon cell and/or the immune cell, the AOM/DSS model may be assessed in mice, by transplanting bone marrow from wild type and knockout animals into irradiated mice of the opposite genotype [68].

Finally, not all colorectal cancer subtypes in humans are linked to inflammation, as our recent findings in human colorectal carcinoma cells showed that NFE2L3 promotes colon cancer cell proliferation in vitro regardless of the immune context. In upcoming studies, it will be of interest to investigate the role of NFE2L3 in other colorectal cancer models, such as the Apc^Min/+^ mice [69], to gain a better understanding of NFE2L3 function in the pathogenesis of various CRC molecular subtypes.

Taken together, we propose that NFE2L3 promotes CRC through changes in the tumor microenvironment, by suppressing the activity of Tregs and through the activation of mast cells, mediated, at least in part, via the increase of IL33 expression and decrease of RAB pathway signaling. Our novel insights into inflammation-induced colorectal cancer development may help in providing novel therapeutic tools for increasing the efficiency of immune checkpoint inhibitor therapies and the prevention of early onset colorectal cancer.

## Materials and methods

### Mouse breeding, treatment, colon, and tumor preparation

Care and handling of animals were in accordance with the federal Health of Animals Act, as practiced by McGill University and the Lady Davis Institute for Medical Research. The mice were littered in aspen shavings and fed regularly (rodent diet 5075, Charles River) with water ad libitum. Offspring from *Nfe2l*3^+/–^ mice (FVB background backcrossed for more than ten generations) mating was used for the AOM/DSS experiment. Heterozygote *Nfe2l3*^+/–^ breeders were inbred to obtain wild type, heterozygote, and homozygote mice. A total of 32 mice were used for the AOM/DSS group: 9 wild type mice (5 males and 4 females), 12 heterozygous mice (6 males and 6 females) and 11 knockout mice (5 males and 6 females). At an adult age (8–14 weeks old), mice received a single dose of azoxymethane (7 mg/kg; Sigma-Aldrich, A5486) by intraperitoneal injection. At week 2, dextran sodium sulfate (2.5%; mpbio, 0216011050) was added to their drinking water for 4 days followed by 17 days of rest. The latter step was repeated two more times for a total of 3. At week 11, mice were sacrificed; entire colons were removed, rinsed with PBS, and opened longitudinally on strips of PBS soaked Whatman filter paper. The whole colon was photographed together with a ruler to determine its size. Normal colon and tumor tissues were dissected and kept in RNA*later* (ThermoScientific, AM7020) to prevent RNA degradation. We determined the number, size and position of tumors using ImageJ software.

### Histological analysis

Following tumor extraction, colons were fixed in 10% formalin, dehydrated in ethanol, embedded in paraffin and 4 µm sections were stained with hematoxylin/eosin. Stained sections were randomized and scored blindly by a board-certified pathologist (Louis Gaboury, IRIC) for presence and intensity of surrogate histological markers of inflammation. Nine such categories were scored on a grading scale of 0–4: inflammatory cell infiltration, inflammatory cell depth, mucosal thickening, surface epithelial degeneration, gland epithelial apoptosis, gland degeneration/abscesses, gland goblet/enterocyte ratio decrease, gland loss, and submucosal edema. Total scores (0–36) combining all nine categories as well as injury scores (0–12) combining scores of inflammatory cell infiltration, surface epithelial degeneration and gland loss, were calculated as previously described [27]. For detection of mast cells, 4 µm paraffin embedded sections were stained with toluidine blue. Mast cells were identified by their purple color and irregular shape [70] and counted blindly using ImageScope.

### DSP/nanostring analysis and immunofluorescence

Digital spatial profiling (DSP) analysis was performed by NanoString’s DSP technology platform to analyze proteins distributed on Formalin-Fixed Paraffin-Embedded (FFPE) tissue sections using the full mouse panel (NanoString Technologies). In brief, 4–6 μm thick FFPE sections were stained for Tryptase (red), PanCK (green), CD45 (yellow), and DNA (blue) to detect ROIs. Area normalization was applied between ROIs of different sizes. This was followed by data normalization using anti rat or rabbit antibodies and the use of three reference proteins including GAPDH, S6 ribosomal protein and histone H3. Results are displayed as absolute expression counts normalized to negative IgG controls. The heatmap was generated using R package pheatmap [71]. Nanostring performed the FOXP3 and CD3 immunofluorescence staining using proprietary antibodies. Staining was performed on wild type (*n* = 5) and knockout (*n* = 5) mice different than the ones stained for DSP.

### Tissue homogenization and RNA extraction

Tissues were removed from RNA*later* and snap-frozen in liquid nitrogen. Once hardened, tissues were homogenized in Trizol reagent (15596018; InVitrogen) using the homogenizer Polytron PT10-35 (Kinematica). Total RNA was extracted using Aurum™ Total RNA Mini Kit (Bio-Rad Laboratories Ltd., Mississauga, Canada). The cDNA was prepared using EasyScript Plus™ cDNA Synthesis Kit (Abmgood Applied Biological Materials Inc., Richmond, Canada) according to the manufacturer’s instructions.

### Reverse transcription quantitative PCR

Total RNA was extracted using Aurum™ Total RNA Mini Kit (Bio-Rad Laboratories Ltd., Mississauga, Canada). The cDNA was prepared using EasyScript Plus™ cDNA Synthesis Kit (Abmgood Applied Biological Materials Inc., Richmond, Canada) according to the manufacturer’s instructions. Transcript abundance was determined by RT-qPCR using SsoAdvanced SYBR Green supermix (Bio-Rad) with the following primers purchased from Biorad: *Il33* (qMmuCID0012110) and *Sytl2* (qMmuCED0047428). Custom primer sequences are listed in Table S2. The RT-qPCR analysis was performed in a CFX96 Touch™ Real Time PCR detection system (Bio-Rad). Data were analyzed by the threshold cycle (Ct) comparative method. Samples were normalized to reference genes *Tbp*, *Rplp0, Nono* and *Eef2*.

### RNA-seq and bioinformatics

Library preparation was performed at McGill University and Génome Québec Innovation Centre. Ribosomal RNA was depleted using NEBNext rRNA Depletion Kit (Human/Mouse/Rat) (E6310, E6350). RNA was bioanalyzed and all samples had a RIN score > 8.5. Sequencing was performed using QCNovaSeq6000 S4 PE100 with an average of 50M reads per sample. RNA sequencing reads were trimmed using Trimmomatic (v0.32), removing adapter and other Illumina-specific sequences as well as the first four bases from the start of each read, and low-quality bases at the end of each read, using a 4 bp sliding window to trim where average window quality fell below 30 (phred33 < 30). Trimmed reads with less than 30 bases were discarded. The resulting clean sets of reads were then aligned to the reference mouse genome build mm10 (GRCm38) using STAR (v2.3.0e) with default parameters. Reads mapping to more than 10 locations in the genome (MAPQ < 1) were discarded.

Gene expression levels were estimated by quantifying uniquely mapped reads to exonic regions (the maximal genomic locus of each gene and its known isoforms) using featureCounts (v1.4.4) and the Ensembl gene annotation set. Normalization (mean of ratios) and variance-stabilized transformation of the data were performed using DESeq2 (v1.14.1). Multiple control metrics were obtained using FASTQC (v0.11.2), SAMtools (v0.1.19) [72], BEDtools (v2.17.0) and custom scripts. Heatmap was generated using R package pheatmap [71].

### HMP2, CIBERSORT, and gene ontology analysis

HMP2 host transcriptomics counts file was used to generate a box plot of NFE2L3 transcript counts using R package ggplot2. CIBERSORT [29] analysis was performed using default parameters, RNAseq gene names were converted to human using biomaRt [73, 74] and compared to the LM22 [75] leukocyte signature matrix. Pathway enrichment analysis was performed using R packages rWikiPathways [76] and clusterProfiler [77] with default settings.

### BMMC generation

BMMC cells were derived as previously reported [31]. Briefly, mouse femur and tibia were isolated and flushed with PBS. The flushed cells were then cultured for 10 passages in RMPI media supplemented with 2-Mercaptoethanol (1:1000, ref), MEM Non-Essential Amino Acids Solution (1:100, Invitrogen 11140050), Sodium pyruvate (1:100, Life Technologies 11360070), HEPES (1:50, Life Technologies 15630-080), Pen Strep (1:100, Invitrogen 15070-063), Newborn calf serum iron-fortified, (10%, Wisent 075-350). Mast cell presence was assessed using Kimura dye [31] and cells were assayed once >95% were stained purple.

### Cell lysis and immunoblot analysis

Whole‐cell extracts were prepared by scraping cells using 1× PBS and cells were lysed for 25 min in whole‐cell lysis buffer (10 mmol/L Tris‐HCl pH 8.0, 420 mmol/L NaCl, 250 mmol/L sucrose, 2 mmol/L MgCl_2_, 1 mmol/L CaCl_2_, 1% Triton‐X100) supplemented with complete protease inhibitor cocktail (Roche, Mississauga, Canada, 04 693 116 001), and then centrifuged at 15,000 *g* for 10 min at 4 °C. Supernatants were collected and protein concentrations were determined using a protein assay kit (Bio‐Rad, 500‐0006). Thirty micrograms of total protein lysate were separated by electrophoresis on Criterion XT Bis-Tris 4-12% (345-0125) and transferred to a polyvinylidene difluoride membrane (Millipore). Blots were blocked using 5% milk in TBST (500 mmol/L Tris pH 7.6, 2 mol/L NaCl, 0.5% Tween) at room temperature for at least 1 h and then incubated overnight at 4 °C with primary antibodies specific for RAB27A (1:200; Abcam ab55667) or ACTIN (1:50,000; Sigma A5441) or Horseradish‐peroxidase (HRP)‐conjugated antibodies were used for 1 h at room temperature. A goat anti-mouse secondary (1:30,000; Thermo Scientific, 31430) was used to detect ACTIN and RAB27A; The antigen‐antibody complexes were visualized using the chemiluminescent HRP substrate (Millipore, WBKLS0500) following the manufacturer’s instructions and exposed to Hyperfilm (GE Healthcare, Baie-d’Urfé, QC, Canada, 28‐9068‐35).

### Chromatin immunoprecipitation

We generated an expression vector coding for the nuclear form of NFE2L3 by mutating the pcDNA3.1-hNFE2L3-V5HIS using Quikchange II XL (Agilent, 200522). HCT116 were transfected 24 h before collection with pcDNA3.1-hNFE2L3 nuclear form-V5His or empty pcDNA3.1-V5His construct using lipofectamine 2000 (ThermoFisher Scientific, 11668027). ChIP assays were carried out using SimpleChIP^®^ Enzymatic Chromatin IP Kit (Cell Signaling Technology, Inc., Danvers, MA, USA, 9003) according to the manufacturer’s instructions. 3 × 10^6^ HCT116 cells were used per IP. Briefly, cells were cross‐linked using 1% formaldehyde for 20 min at room temperature and quenched with glycine to a final concentration of 0.125 mol/L for 5 min. Subsequently, DNA was lysed for 20 min at 37 °C using 0.4 μL of micrococcal nuclease (Cell Signaling, 10011). Digestion was stopped by adding 10 μL of 0.5 mol/L EDTA (Cell Signaling, 7011) per IP prep on ice for 2 min. This was followed by nuclei lysis with three sets of 20‐second pulses at 15% intensity using a Fisher Scientific model 500 sonic dismembrator. The samples were then incubated overnight with ChIP‐Grade Protein G Magnetic Beads (Cell Signaling, 9006) with an antibody specific for NFE2L3, we had generated previously [67]. The corresponding homemade preimmune rabbit IgG was used as a negative control. After stringent washes, chromatin was eluted in ChIP Elution Buffer (Cell Signaling, 7009). Eluates were reverse cross‐linked by adding 6 μL 5 mol/L NaCl and 2 μL Proteinase K (Cell Signaling, 10012), and incubated for 2 h at 65 °C. DNA was extracted and purified using Spin Columns (Cell Signaling, 10010) and eluted in DNA Elution Buffer (Cell Signaling, 10009). ChIP‐qPCR analyses were performed in a CFX96 Touch™ Real Time PCR detection system (Bio‐Rad) and the primers used are listed in Table S2. Primers for *RPL30* were used as a negative control and showed no significant enrichment upon IP (Cell signaling, Primers #7014).

### Statistical analysis

*P* values were calculated with an unpaired Student’s *t* test. *P* values of 0.05 or less were considered significant, *p ≤ 0.05; **p ≤ 0.01; ***p ≤ 0.001 and ****p ≤ 0.0001 and ^ΔΔ^*p* ≤ 0.01 and ^ΔΔΔ^*p* ≤ 0.001. Statistical analysis was performed using R functions “stat_compare_means” and “compare_means” using default conditions (*t*-test unpaired); Figures were generated using R package “ggplot2” [78].

## Supplementary information

Supplementary Information

Supplementary table 1

Supplementary table 2

Supplementary Figures
