# Supplementary material for: Loss of NFE2L3 protects against inflammation-induced colorectal cancer through modulation of the tumor microenvironment
Source: Oncogene. 2022 Jan 28;41(11):1563–75. doi: 10.1038/s41388-022-02192-2 (PMC8913363; doi:10.1038/s41388-022-02192-2)
Supplement: Supplementary file 1 — Supplementary Information [file 41388_2022_2192_MOESM1_ESM.doc]

**Suppl. Table 1 Pathway enrichment analysis of tumor derived RNA-seq data**Pathway enrichment analysis of RNA-seq data derived from *Nfe2l3* knockout tumors compared to wild type tumors. Data is sorted by odds ratio.

**Suppl. Table 2 Primers for RT-qPCR and ChIP-qPCR analysis**

**Suppl. Fig. 1 Loss of NFE2L3 leads to a decrease in tumor size and injury score.
a** Boxplot representation of tumor surface distribution per genotype. **b** Quantification of tumor distribution per section of the colon, including distal, mid, and proximal location. **c, d** Boxplot representation of colon injury score per genotype in full length **c** or distal, mid, and proximal sections **d** scored from 0 to 4 (0-absence; 1-very mild/debatable; 2-mild; 3-moderate; 4-severe) using inflammatory cell infiltration, surface epithelial degeneration and gland loss criteria. *T-*test was performed for statistical analysis. Data represents unpaired *t-*test; **p* ≤ 0.05; ***p* ≤ 0.01; ****p* ≤ 0.001 and *****p* ≤ 0.0001; and mean values ± SD.

**Suppl. Fig. 2 NFE2L3 transcript levels are elevated in ulcerative colitis patients**Boxplot representation of NFE2L3 mRNA transcript levels derived from RNA-seq data of the HMP2 project (28) of the lower intestine, colon and rectum from individuals not diagnosed with inflammatory bowel disease (nonIBD), Crohn’s disease (CD) or Ulcerative colitis (UC). *T-*test was performed for statistical analysis. Data represents unpaired *t-*test; ***p* ≤ 0.01 and ****p* ≤ 0.001.
