# Supplementary material for: Loss of NFE2L3 protects against inflammation-induced colorectal cancer through modulation of the tumor microenvironment
Source: Oncogene. 2022 Jan 28;41(11):1563–75. doi: 10.1038/s41388-022-02192-2 (PMC8913363; doi:10.1038/s41388-022-02192-2)
Supplement: Supplementary file 3 — Supplementary table 2 [file 41388_2022_2192_MOESM3_ESM.pdf]

Table S2

a) Primers for RT-qPCR

Mus Musculus gene name	Forward and reverse primer sequence
Eef2	Fwd: 5’-TGTCAGTCATCGCCCATGTG-3’
	Rev: 5’-CATCCTTGCGAGTGTCAGTGA-3’
Il33	Purchased from Biorad (qMmuCID0012110)
Myrip	Fwd: 5’- AGCCCTGTCTTCACTTCCAAG-3’
	Rev: 5’- AGCAGCAATGGCAAACAAGG-3’
Nfe2l3	Fwd: 5’-ACAGCCTGAACATTCTCTGGA-3’
	Rev: 5’-TGGCTTATAGCTTGGCTCAAA-3’
Nono	Fwd: 5’-AAAGCAGGCGAAGTTTTCATTC-3’
	Rev: 5’-ATTTCCGCTAGGGTTCGTGTT-3’
Rab27a	Fwd: 5’-TTTCGGAGCTTAACCACTGC-3’
	Rev: 5’-TTTCACAGTACGCGTGCATC-3’
Rab27b	Fwd: 5’- AGTCAACTGCAGGCAAATGC-3’
	Rev: 5’- TTGCCGTTCATTGACTTCCC-3’
Rplp0	Fwd: 5’-AGATTCGGGATATGCTGTTGGC-3’
	Rev: 5’-TCGGGTCCTAGACCAGTGTTC-3’
Tbp	Fwd: 5’-CCAATGACTCCTATGACCCCTA-3’
	Rev: 5’-CAGCCAAGATTCACGGTAGAT-3’
Sytl2	Purchased from Biorad (qMmuCED0047428)
Sytl4	Fwd: 5’- TGGAGCTAGAGACCCTTCCA-3’
	Rev: 5’- TCAAATCCCTTTCCATCTCG-3’

b) Primers for ChIP-qPCR

Homo sapiens gene name	Forward and reverse primer sequence
Il33 peak A	Fwd: 5’-CTTGCATCTACCCTGAAAGGAG-3’
	Rev: 5’-TGGTTACCCCAGGTTTTGTG-3’
Il33 peak B	Fwd: 5’-TGGGCACACTGATATTGCTG-3’
	Rev: 5’-AGGGGAAAGGAGACACAAAC-3’
Rab27a peak A	Fwd: 5’- ATTCCCTCGCTTGTGTATGG-3’
	Rev: 5’- TAACTTTCCTGGCCATCTGC-3’
Rab27a peak B	Fwd: 5’-GGTAGTGAACCTGGGACTGC-3’
	Rev: 5’-ACTGAGAGGGATGAACTTGGCC-3’
Rab27b peak A	Fwd: 5’- ATACCTGCCTGAGTTTCCACAG-3’
	Rev: 5’- AAAGCTCTCACTGTGCCAAC-3’
Rab27b peak B	Fwd: 5’- TTGGCCAAACTCAGTGACTG-3’
	Rev: 5’- AGTTCAGCCTAAAGGTTTCTCTG-3’
RPL30	Purchased from Cell signaling technology
	(SimpleChIP® Human RPL30 Exon 3 Primers #7014)
