# Supplementary figures and images for: Loss of NFE2L3 protects against inflammation-induced colorectal cancer through modulation of the tumor microenvironment
Source: Oncogene. 2022 Jan 28;41(11):1563–75. doi: 10.1038/s41388-022-02192-2 (PMC8913363; doi:10.1038/s41388-022-02192-2)

Supp. Figure 1

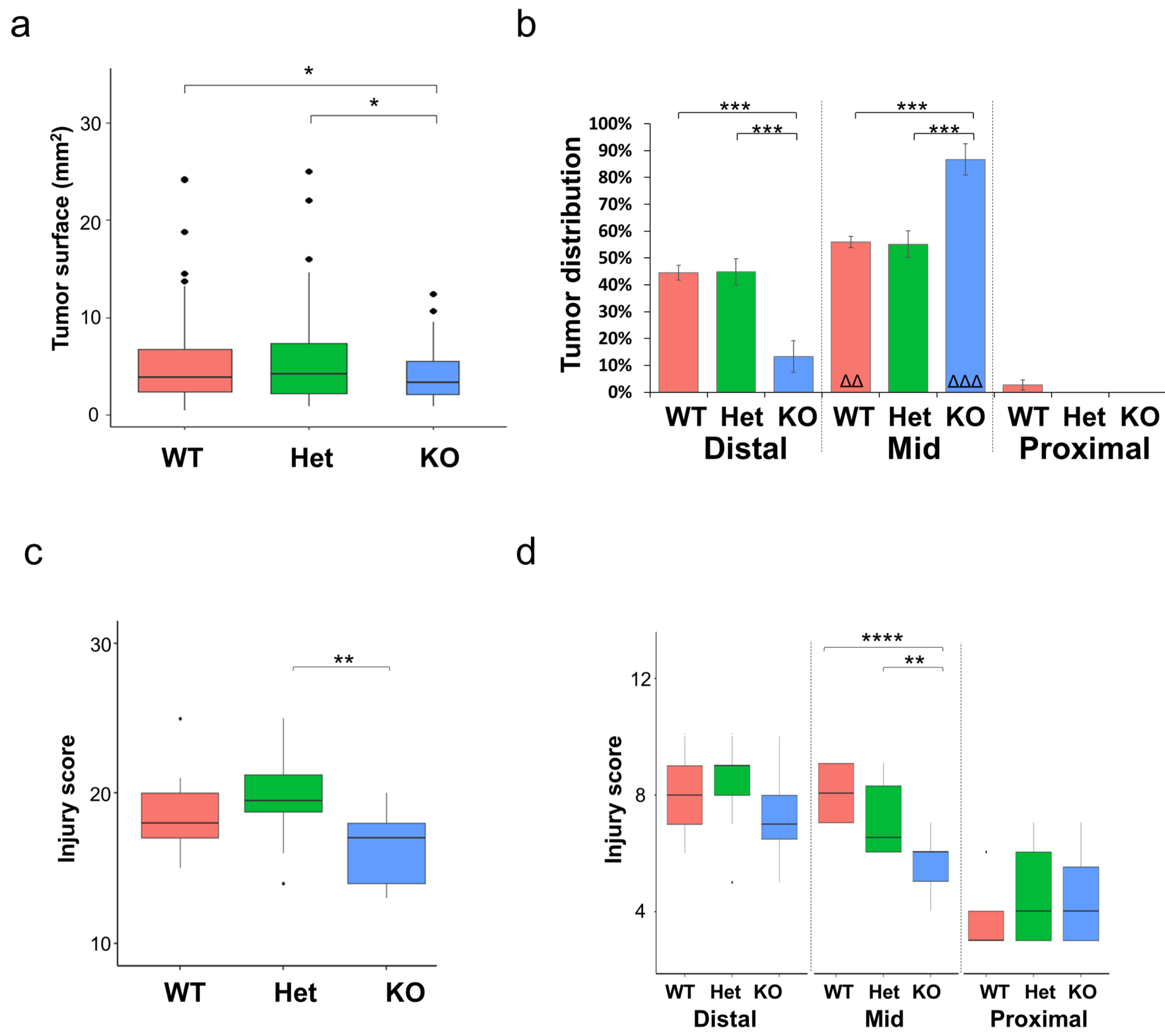

Supp. Figure 2

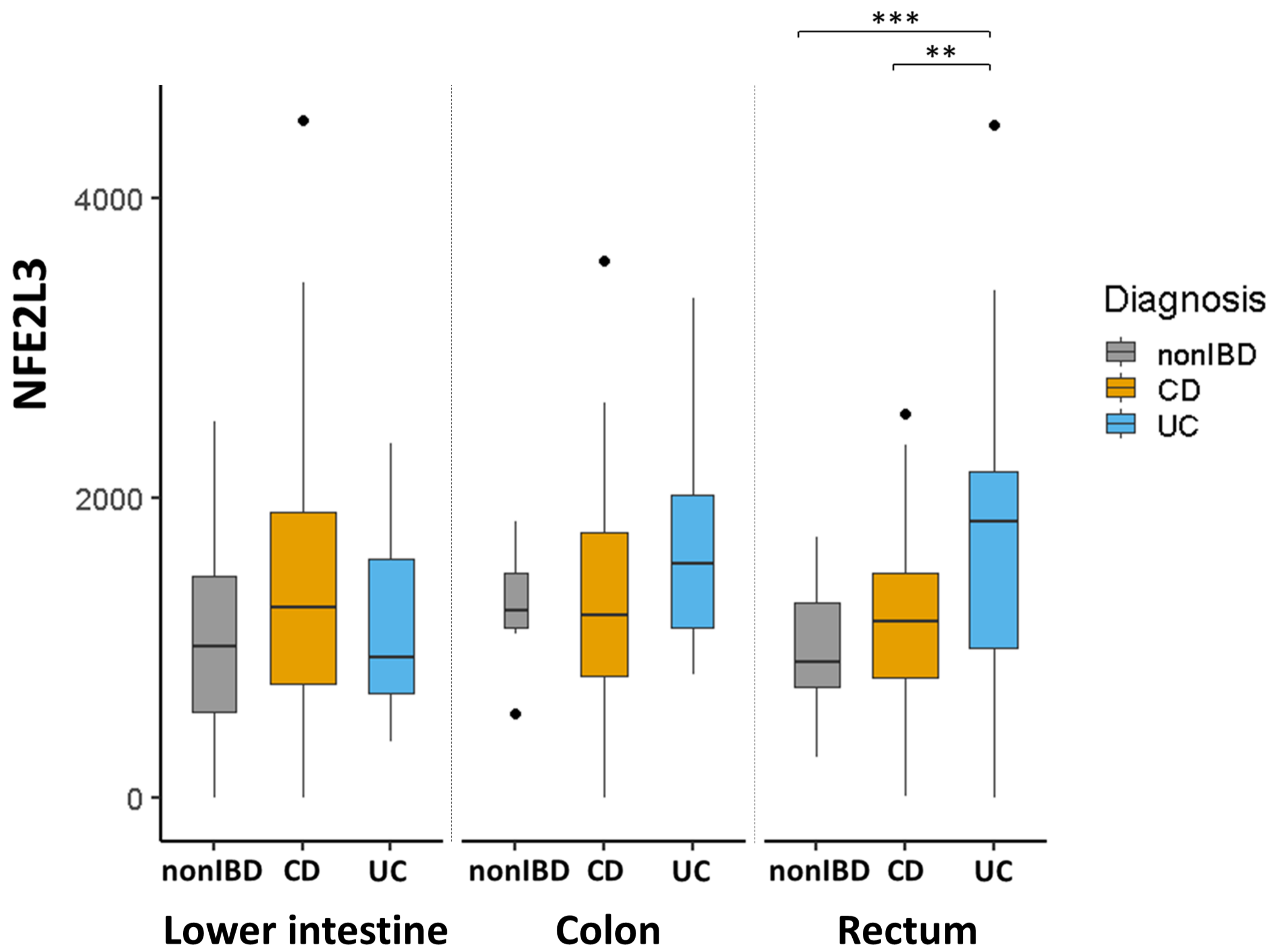

Supplement: Supplementary file 4 — Supplementary Figures [file 41388_2022_2192_MOESM4_ESM.pdf]
